# Iron retardation in lysosomes protects senescent cells from ferroptosis

**DOI:** 10.18632/aging.205777

**Published:** 2024-04-26

**Authors:** Yujing Feng, Huaiqing Wei, Meng Lyu, Zhiyuan Yu, Jia Chen, Xinxing Lyu, Fengfeng Zhuang

**Affiliations:** 1 School of Laboratory Animal and Shandong Laboratory Animal Center, Shandong First Medical University and Shandong Academy of Medical Sciences, Jinan, Shandong, China; 2 School of Clinical and Basic Medical Sciences, Shandong First Medical University and Shandong Academy of Medical Sciences, Jinan, Shandong, China; 3 Biomedical Research College and Shandong Medicinal Biotechnology Centre, Shandong First Medical University and Shandong Academy of Medical Sciences, Jinan, Shandong, China; 4 School of Radiology, Shandong First Medical University and Shandong Academy of Medical Sciences, Taian, Shandong, China

**Keywords:** iron accumulation, senescent cells, lysosome, ferroptosis, ferritinophagy

## Abstract

Ferroptosis, an iron-triggered modality of cellular death, has been reported to closely relate to human aging progression and aging-related diseases. However, the involvement of ferroptosis in the development and maintenance of senescent cells still remains elusive. Here, we established a doxorubicin-induced senescent HSkM cell model and found that both iron accumulation and lipid peroxidation increase in senescent cells. Moreover, such iron overload in senescent cells has changed the expression panel of the ferroptosis-response proteins. Interestingly, the iron accumulation and lipid peroxidation does not trigger ferroptosis-induced cell death. Oppositely, senescent cells manifest resistance to the ferroptosis inducers, compared to the proliferating cells. To further investigate the mechanism of ferroptosis-resistance for senescent cells, we traced the iron flux in cell and found iron arrested in lysosome. Moreover, disruption of lysosome functions by chloroquine and LLOMe dramatically triggered the senescent cell death. Besides, the ferroitinophagy-related proteins FTH1/FTL and NCOA4 knockdown also increases the senescent cell death. Thus, we speculated that iron retardation in lysosome of senescent cells is the key mechanism for ferroptosis resistance. And the lysosome is a promising target for senolytic drugs to selectively clear senescent cells and alleviate the aging related diseases.

## INTRODUCTION

Senescence presents a unique cellular fate in multicellular organisms, characterized by a variety of hallmarks of irreversible cell cycle arrest, senescence-associated secretory phenotype (SASP), genome instability and many other unique morphologies [1]. Cellular senescence is ubiquitously elicited by a variety of environmental or intrinsic stressors such as ultraviolet irradiation, genotoxins, oxidative stress, mitochondrial dysfunction, oncogene activation and/or telomere attrition [2–7]. The extensive accumulation of senescent cells in mitotic tissues is a typical biological feature for aging and recognized as a vital causative factor for the development of age-related diseases [8]. On the other hand, cellular senescence also has beneficial functions, such as embryonic development, tumor suppression, wound healing and immune response [9–11]. Over the past decades, substantial studies have also been focused on the unique processes of cellular senescence, and seek for possible strategies to intervene geriatric diseases and increase healthy lifespan via specific eliminating the number of senescent cells [12].

With aging, iron accumulation has been found in multiple human organs, which has been reported to be associated with late-life illnesses including Alzheimer Parkinson, hyperpigmentation, diabetes mellitus [8]. Generally, cellular iron homeostasis is strictly regulated by multiple iron metabolic processes, involving the iron uptake, storage and efflux, closely related to organism aging and longevity. In fact, the dysfunction of iron metabolism for senescent cells is a major causative factor to the detrimental iron accumulation in aging tissues [13]. Transferrin receptor 1 (TfR1), a transmembrane glycoprotein mediating the cellular iron uptake, has been found to be overexpressed in IR-induced senescent MEF cells [14]. And even more than ten-fold increase of intracellular iron concentration has been reported in both replicative and stress-induced senescent human fibroblast cells [15]. The overloaded iron is a risk to induce ferroptosis, one of the programmed cell death processes caused by the accumulation of lipid peroxidation via Fenton reaction. Meanwhile, senescent cells also manifest high oxidative stress. Even though senescent cells exhibit both the two typical characterizations of ferroptosis, iron accumulation and lipid peroxidation, they are still able to sustain metabolic viability for more than several months [16]. Besides, senescent cells even show a strong ferroptosis resistance under ferroptosis-inducer treatment [14]. However, the mechanism of ferroptosis resistance in senescent cells still remains elusive.

Iron metabolism has been reported to play a vital role in muscle functions by participating in variety of biological processes, including oxygen supply, energy production and muscle protein synthesis and degradation [17]. While with aging, skeletal muscle will gradually lose mass, strength, and also the self-regenerative ability. Growing evidence suggested that dysregulation of iron metabolism plays a role in age-related skeletal muscles disease, sarcopenia [18]. In aged rat model, free iron ions have been found to accumulate in skeletal muscle [19]. Therefore, iron-dependent ferroptosis attracts enormous attention on muscle aging and relevant diseases.

Clearance of senescent cells has been reported to benefit the extend of healthy life and alleviate the aging-related diseases [20]. The aberrant iron accumulation and lipid peroxidation in senescence cells implicated that ferroptosis-response pathway might be a promising senolytic target [21]. To investigate the cellular processes in response to ferroptosis in senescent cells, studies of the expression modality of ferroptosis-response proteins and autophagy-associated cellular processes in senescent cells are included in this study. We first established the senescent HSkM cell model and validated that doxorubicin (Dox) induced senescent HSkM cells manifested iron accumulation and high level of lipid peroxidation. While senescent HSkM cells are resistant to the GPX4 inhibitors, RSL3 and FIN56, and also the FSP1 inhibitor iFSP1. Iron trace indicates that most of the iron is arrested in lysosomes, and the senescent cells are more sensitive to lysosomotropic drugs of chloroquine (CQ) and L-Leucyl-L-Leucine methyl ester (LLOMe). Thus, we proposed that the increased lysosomes in senescent cells function to control the release of the iron, and then avoid the overloaded iron-induced ferroptosis.

## MATERIALS AND METHODS

### Antibodies and reagents

The antibodies GPX4 (Cat# ab125066), LC3 (Cat# ab48394), NCOA4 (Cat# ab86707) were purchased from Abcam. The antibodies SLC7A11 (Cat# 12691), P16 (Cat# 80772), P21 (Cat# 2947) were purchased from Cell Signaling Technology. The antibodies ACSL4 (Cat# A20414), FTH1 (Cat# A19544), FSP1 (Cat# A12128), DMT1/SLC11A2 (Cat# A23379), CD71/Transferrin (Cat# A21622), SLC40A1 (Cat# A14884) were purchased from Abclonal. The antibodies P62 (Cat# 18420-1-AP), GAPDH (Cat# 60004-1-lg), Ki67 (Cat# 27309-1-AP). The BODIPY™ 581/591 C11 (Cat# D3861), LysoTracker (Cat# L7526) were purchased from Thermo Fisher Scientific. The FerroOrange (Cat# F374) was purchased from Dojindo. The ferroptosis inducers, RSL3 (Cat# SML2234) was purchased from Merck; Fin56 (Cat# HY-103087) and iFSP1 (Cat# HY-136057) were purchased from MCE. The chloroquine (Cat# HY-17589A), LLOMe (Cat# HY-129905A) were purchased from MCE. Doxorubicin hydrochloride (Cat# D807083) was purchased from Macklin.

### Cell culture

Human skeletal myoblast (HSkM) cells were cultured in Dulecco’s modified Eagle’s medium (DMEM) (Cat# L110KJ, BasalMedia) supplemented with 10% FBS (Cat# S711-001S) and 100 U penicillin, 100 μg/ml streptomycin (Cat# P1400, Solarbio).

### Senescent HSkM cells induction

Senescent HSkM cells were induced by doxorubicin. HSkM cells were cultured until the cell confluency reached about 60% on dishes, and then treated with 50 nM doxorubicin for three days, and then removed the drug and continue to culture the cells for another three days.

### Senescence-associated β-galactosidase assay

SA-β-gal staining was performed using the Cell Senescence β-galactosidase Staining kit (Cat# C0602, Beyotime) according to the manufacturer’s instructions. Images were taken with an inverted light microscope (ECHO).

### Immunofluorescence (IF) staining

Immunofluorescence staining was performed as described previously [22]. Briefly, cells were cultured on cover slips and then fixed with 4% paraformaldehyde (PFA) for 15 min at room temperature. Following fixation, cells were permeabilized with 0.15% Triton X-100 in PBS for 15 min, and washed 3 times for 5 min with PBS. Next, samples were blocked with 1% BSA in PBS for 60 min at room temperature and then incubated with primary antibodies overnight at 4°C. Then cells were washed three times with PBS and incubated with secondary antibodies for 1 h at room temperature. The cells were washed 3 times and stained with DAPI for 10 min at room temperature. The cell cover slips were sealed with an anti-fluorescence quench agent and imaged using microscopy (ECHO).

### Proliferating cells detection (BrdU staining under the denature condition)

Proliferating cells were detected through the detection of BrdU incorporation during DNA synthesis. Briefly, cells were incubated for 40 min in medium containing 10 μM BrdU, and then washed twice with PBS. Subsequently, cells were fixed with 4% paraformaldehyde for 15 min at room temperature, and washed three times with PBS. Next, the fixed cells were incubated with 1M HCl at 37°C for 20 min to denature the duplex DNA, and the cells were washed three times with PBS. Finally, regular IF procedures were then followed with BrdU primary antibodies (see as Immunofluorescence (IF) staining). The coverslips were blocked with an anti-fluorescence quench and imaged by microscopy.

### Quantitative real-time polymerase chain reaction (qPCR) analysis

Total RNA were isolated using SPARKeasy Tissue/Cell RNA kit (Cat# AC0205-A, SparkJade) and reverse transcription to cDNA were performed by the FastKing cDNA first strand Synthesis kit (Cat# KR116, TIANGEN). Quantitative real-time PCR was running on a LightCycler^®^ 96 Instrument (Roche).

### Western blotting analysis

Cells were lysed in RIPA buffer, and the lysate was centrifuged to remove the insoluble fraction. Protein concentration was estimated BCA method by BCA kit (Cat# 20201ES76, Yeasen). Equal protein amount of sample was loaded per lane, and subjected to SDS-PAGE. The samples were transferred to the PVDF membrane. Subsequently, the blots were blocked for 1 hour, and then incubated with respective primary antibodies overnight at 4°C. Peroxidase-conjugated secondary antibodies were used corresponding to their primary antibody. Signals were normalized using housekeeping protein GAPDH. The blot was developed using ECL reagent (Cat# SQ101, EpiZyme), and signal was visualized on automatic chemiluminescence image analysis system (Tanon 5200).

### Detection of Fe^2+^, lipid oxidation and lysosome

The Fe^2+^ probes FerroOrange (Dojindo, F374), C11-BODIPY581/591 (Thermo Fisher Scientific™, D3861) and LysoTracker™ Green DND-26 (Thermo Fisher Scientific™, L7526) and Hoechst 33342 (Thermo Scientific™, R37605) were used to detect the intracellular free Fe^2+^, lipid oxidation, lysosome and nuclei respectively. Cells were plated on 8-well Chambered Coverglass (Thermo Fisher Scientific™ Nunc™ Lab-Tek™ II), and stained with the probes as indicated in each experiment at 37°C, then imaged on a Zeiss Celldiscoverer 7 inverted microscope using corresponding filters. The images were analyzed in the software of ImageJ.

### Gene knockdown by small interfering RNA (siRNA)

The siRNA target sequence was prepared as follows: human NCOA4 forward 5′-ACUCUUGUUUAUCGAAGUAUATT-3′, reverse 5′-UAUACUUCGAUAAACAAGAGUTT-3′; human FTL forward 5′-CCUGGAGACUCACUUCCUATT-3′, reverse 5′-UAGGAAGUGAGUCUCCAGGAA-3′; human FTH1 forward 5′-GAAUCAGUCACUACUGGAACTT-3′, reverse 5′-GUUCCAGUAGUGACUGAUUCTT-3′; human TFEB siRNA pool (5′-CAGGCUGUCAUGCAUUACATT-3′, 5′-GGCUACAUCAAUCCUGAAATT-3′ and 5′-GACGAAGGUUCAACAUCAATT-3′). Transfections were performed with siRNA-Mate (Cat# G04003, GenePharma) according to the manufacturer’s instructions.

### Malondialdehyde (MDA) assay

The MDA content was determined using a malondialdehyde (MDA) content assay kit, according to the manufacturer’s instructions (Cat# BC0025, Solarbio).

### Cell viability assay

Cell viability was detected by cell counting kit-8 (CCK-8) (Cat# 40203ES76, Yeasen) and crystal violet staining. For CCK-8 assay, cells were seeded in 96-well plates with 100 μL of culture medium. After drug treatment, 100 μL medium containing 10 μL CCK-8 reagent was added to each well at the indicated time points. The plates were incubated at 37°C in the dark for 1 h, and the absorbance at 450 nm was measured using a microplate reader. For crystal violet staining, cells were seeded in 24-well plates. After treatment, the cells were fixed with 4% paraformaldehyde for 15 min, and washed three times with PBS. Subsequently, fixed cells were stained with 0.05% crystal violet for 1 h at room temperature. And the plates will be scanned, and also the cell number will be quantified using microscopy.

### Statistical analysis

Statistics were performed using GraphPad Prism 7. A standard two-tailed unpaired student’s *t*-test, one way ANOVA or two-way ANOVA was used for statistical analysis for specific experiments. All the *p*-values are indicated in the figures.

## RESULTS

### Induction of human HSkM cellular senescence by doxorubicin treatment

**Figure 1:**
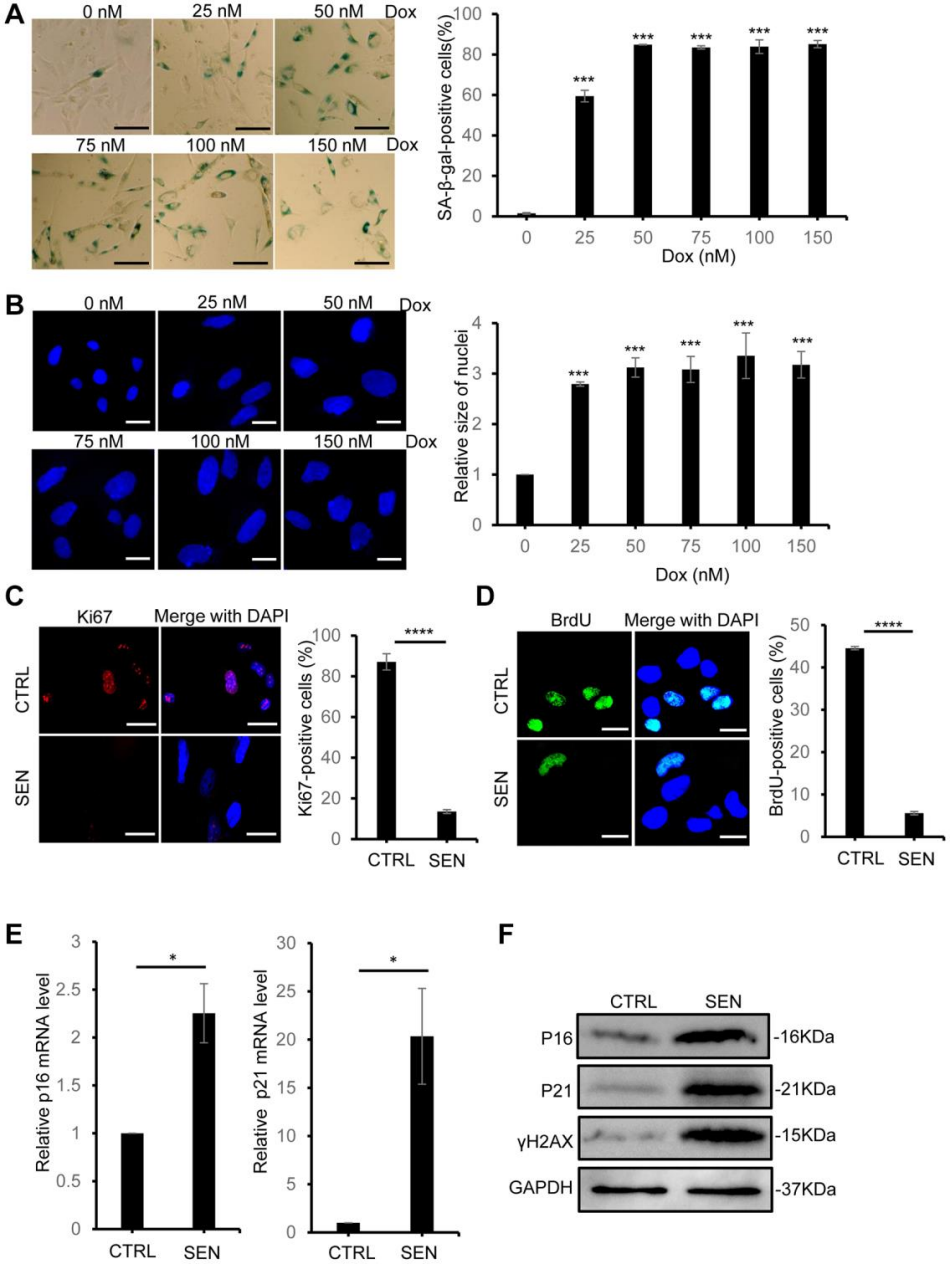
**Doxorubicin treatment induces cellular senescence in HSkM cells.** (**A**) The SA-β-gal activity of HSkM cells were measured with a series of concentrations of doxorubicin treatment. Scale bars: 80 μm. *P*-values were calculated by one-way ANOVA analysis with post hoc Tukey, ^***^P < 0.001. Data represented as mean ± SD. (**B**) Quantification of the nucleus size of HSkM with the indicated concentrations of doxorubicin treatment. Scale bars: 40 μm. *P*-values were calculated by one-way ANOVA analysis with post hoc Tukey, ^***^P < 0.001. Data represented as mean ± SD. (**C**) Immunofluorescence analysis of Ki67 expression in proliferating and Dox-induced senescent HSkM cells. Scale bars, 80 μm. *P*-values were calculated by two-tailed unpaired student’s *t*-test, ^****^P < 0.0001. Data represented as mean ± SD. (**D**) BrdU incorporation analysis in proliferating and senescent HSkM cells. Scale bars, 20 μm. *P*-values were calculated by two-tailed unpaired student’s *t*-test, ^****^P < 0.0001. Data represented as mean ± SD. (**E**) qPCR analysis of the transcription of p16 and p21 in proliferating and senescent HSkM cells. *P*-values were calculated by two-tailed unpaired student’s *t*-test, ^*^P < 0.05. Data represented as mean ± SD. (**F**) Western blot analysis of the expression of P16, P21 and γH2AX in proliferating and senescent HSkM cells. All the experiments have been performed for at least 3 independent biological repeats.

Doxorubicin (Dox) is a common natural chemotherapeutic drug to kill cancer cells via disrupting cellular processes, including ROS overproduction, DNA damage and replication stress induction and others. Besides, Dox is also found to induce cellular senescence in multiple cell types and mouse models [23]. Here, we used Dox to build a stress-induced senescent HSkM cell model. First, to efficiently increase the ratio of senescent cell formation but reduce cell death, we tested a series of concentrations of Dox to treat HSkM cells for three days, then removed the drug and continued to culture the cells with fresh medium for another 3 days (Supplementary Figure 1). The efficiency of Dox-induced senescent cells was determined by several well-recognized cellular senescent markers in decades. As shown in Figure 1A, 50 nM Dox treatment can induce more than 85% of HSkM cells to the senescent status via senescence-associated beta-galactosidase (SA-β-gal) activity analysis. And we found that higher concentrations of Dox would not further elevate the senescence-inducing efficiency but raise more cell lethality (Figure 1A). Besides, DAPI staining indicates that the size of nuclei was enlarged and also no further increase after treatment with 50 nM or higher concentration of Dox (Figure 1B). To further validate the efficiency of induced senescent cell with 50 nM Dox treatment, we used the Ki67 staining and BrdU incorporation to label the replicative HSkM cells, and the immunofluorescence results indicate that less than 5% HSkM cells stays in the normal cell cycle (Figure 1C, 1D). Subsequently, we analyzed the expression of two classical senescent marker genes, p16 and p21, and the two genes showed dramatic increase by qPCR and Western blot analysis (Figure 1E, 1F). Meanwhile, another senescence marker, γH2AX (phosphorylation of ser139 on H2AX), also increased in senescent HSkM cells (Figure 1F). Collectively, these results indicated that this Dox-treatment procedure is capable of efficient buildup of the stress-induced senescent HSkM cell model.

### Cellular senescence increases iron accumulation and alters the expression pattern of ferroptosis-response genes

**Figure 2:**
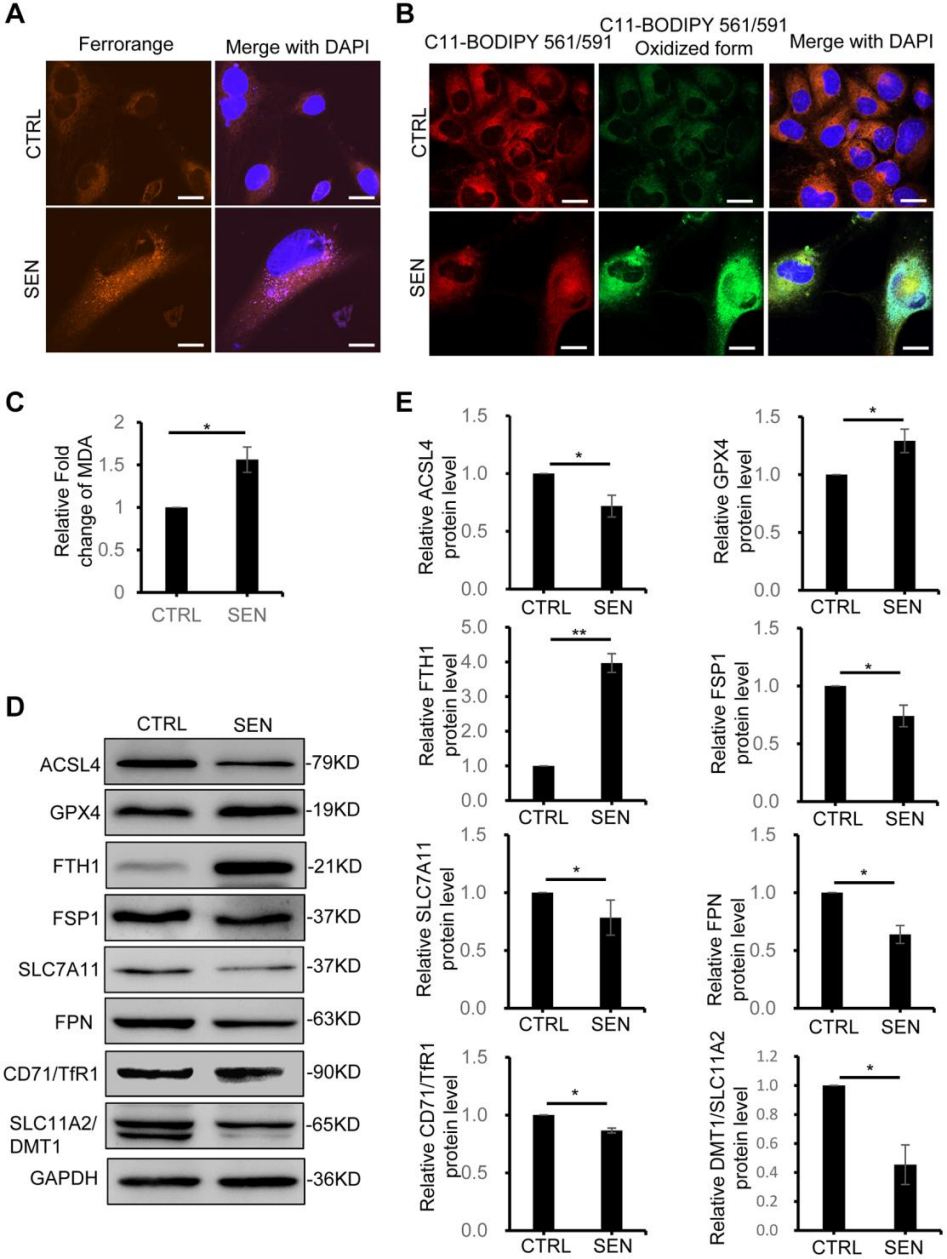
**Dox-induced cellular senescence increases Fe^2+^ accumulation and lipid peroxidation and alters the ferroptosis-related gene expression.** (**A**) Representative live-cell fluorescence images of intracellular Fe^2+^ in proliferating and senescent cells. Fe^2+^ was detected by the probe of FerroOrange. Nuclei was stained with Hoechst 33342. Scale bars, 20 μm. (**B**) Representative fluorescent images of C11-BODIPY 561/591 staining shows the increase of lipid peroxidation in senescent HSkM cells. Red represents reduced BODIPY-C11 signal and Green represents oxidized BODIPY-C11 signal. Nuclei were stained with Hoechst 33342. Scale bars, 20 μm. (**C**) MDA detection assay. The MDA production assay was performed in both proliferation and senescent cells. *P*-values were calculated by two-tailed unpaired student’s *t*-test, ^*^P < 0.05. Data represented as mean ± SD. And three biological experiment repeats have been performed. (**D**, **E**) Western blot analysis of ferroptosis-response protein expression in proliferating and senescent HSkM cells. (**D**) Immunoblot representative images and (**E**) quantification of ACSL4, GPX4, FTH1, FSP1, SLC7A11, SLC40A1, CD71/TFR1 and SLC11A2/DMT1 protein expression. Each protein band intensity was measured and normalized to GAPDH and compared between proliferating and senescent cells. *P*-values were calculated by two-tailed unpaired student’s *t*-test, ^*^P < 0.05, ^**^P < 0.01. Data represented as mean ± SD. And three independent biological experiment repeats have been performed.

Iron homeostasis in tissue and cells plays an important role in cellular metabolic processes, including redox homeostasis, oxidative supply and energy production [24, 25]. Here we found a remarkable increase of iron ions in Dox-induced senescent HSkM cells by Fe^2+^ probe FerroOrange staining (Figure 2A). Usually, the ferroptosis is typically thought to be caused by iron accumulation to increase ROS pool via Fenton reaction, and finally results in lipid peroxidation-mediated cell death [26]. Therefore, we employed the C11-BODIPY561/591 probe to measure the oxidative extent of lipid in senescent cells. Consistent with iron accumulation, oxidative form of C11-BODIPY561/519 probe also dramatically increases in senescent cells (Figure 2B). In the meantime, malondialdehyde (MDA) assay, another well-recognized marker of ferroptosis via measuring production of oxidative stress, also exhibits a 1.6-fold increase in senescent cells (Figure 2C). Thus, the iron accumulation and excess lipid peroxidation suggested that ferroptosis might participate in the induction and/or maintenance of cellular senescence.

To further analysis the molecular processes of how senescent cells response to ferroptosis-related iron accumulation and lipid peroxidation, we analyzed the expression patterns of ferroptosis-associated proteins in senescent HSkM cells (Figure 2D, 2E and Supplementary Figure 2). Currently, four classic pathways have been generally investigated in cells in response to ferroptosis, including system Xc^−^-glutathione-GPX4 axis, FSP1-CoQ10-NADPH, iron metabolism and lipid metabolism [26]. Here, we profiled the changes of several key ferroptosis-related proteins in these pathways under the context of Dox-induced cellular senescence and investigated the possible mechanism underlying the reason why senescent cells can resist ferroptosis. First, as for Xc^−^-glutathione-GPX4 axis, the transcription and expression of Cystine transporter SLC7A11 shows slightly decrease, and glutathione peroxidase GPX4, one important intracellular antioxidant protein to regulate ferroptosis, only shows a slight increase in senescent HSkM cells. Besides, FSP1 mediated CoQ antioxidant system acts parallel to GPX4 to inhibit ferroptosis [27]. We found a three-fold increase of *FSP1* gene transcription, but its protein level decreases in senescent cells, which is possibly caused by translation inhibition or protein degradation of FSP1 in Dox-induced senescent cells. Thus, the above two pathways seem not to be the key factors for senescent cells to response to the iron accumulation. Accordingly, the abnormal iron accumulation in senescent cells suggests the disruption of iron metabolism. We therefore sought the molecular causes for dysfunction of iron homeostasis maintenance in senescent HSkM cells. Both iron transporter protein DMT1 and Transferrin receptor 1 (TFR1), which mediates the iron transporting into cells, did not show increase in senescent HSkM cells. While an iron transporter FPN, which has been reported to promote cellular iron release, decreases in senescent cell. Thus, the decrease of FPN-mediated iron export contributes to iron accumulation in senescent cells, rather than TRF1- and/or DMT-mediated iron import. The decrease of TRF1 and DMT1 expression might be caused by the negative regulation of iron concentration in senescent cells. The cellular iron-storage protein ferritin, composing of two subunits FTH1 and FTL, dramatically increase in senescent cells, which will limit the iron release and redox-active iron production in senescent cells. In addition, dysregulation of lipid peroxidation is the causative mechanism of ferroptosis. In senescent HSkM cells, acyl-CoA synthetase long-chain family member 4 (ACSL4), one of the main factors to promote ferroptosis by contributing to the synthesis of PUFAs, is found to decrease [28]. Except for these classic ferroptosis marker genes, another two recently reported ferroptosis marker genes, CHAC1 and FTGS2, are also upregulated in senescent cells detected by qPCR, similar to those in ferroptosis-induced normal cells (Supplementary Figure 2). Taken together, the changes of expression pattern of ferroptosis-related marker genes indicates that senescent cells response to the iron accumulation and limit cell death caused by the ferroptosis.

### Senescent HSkM cells are resistant to pro-ferroptotic stimuli

**Figure 3:**
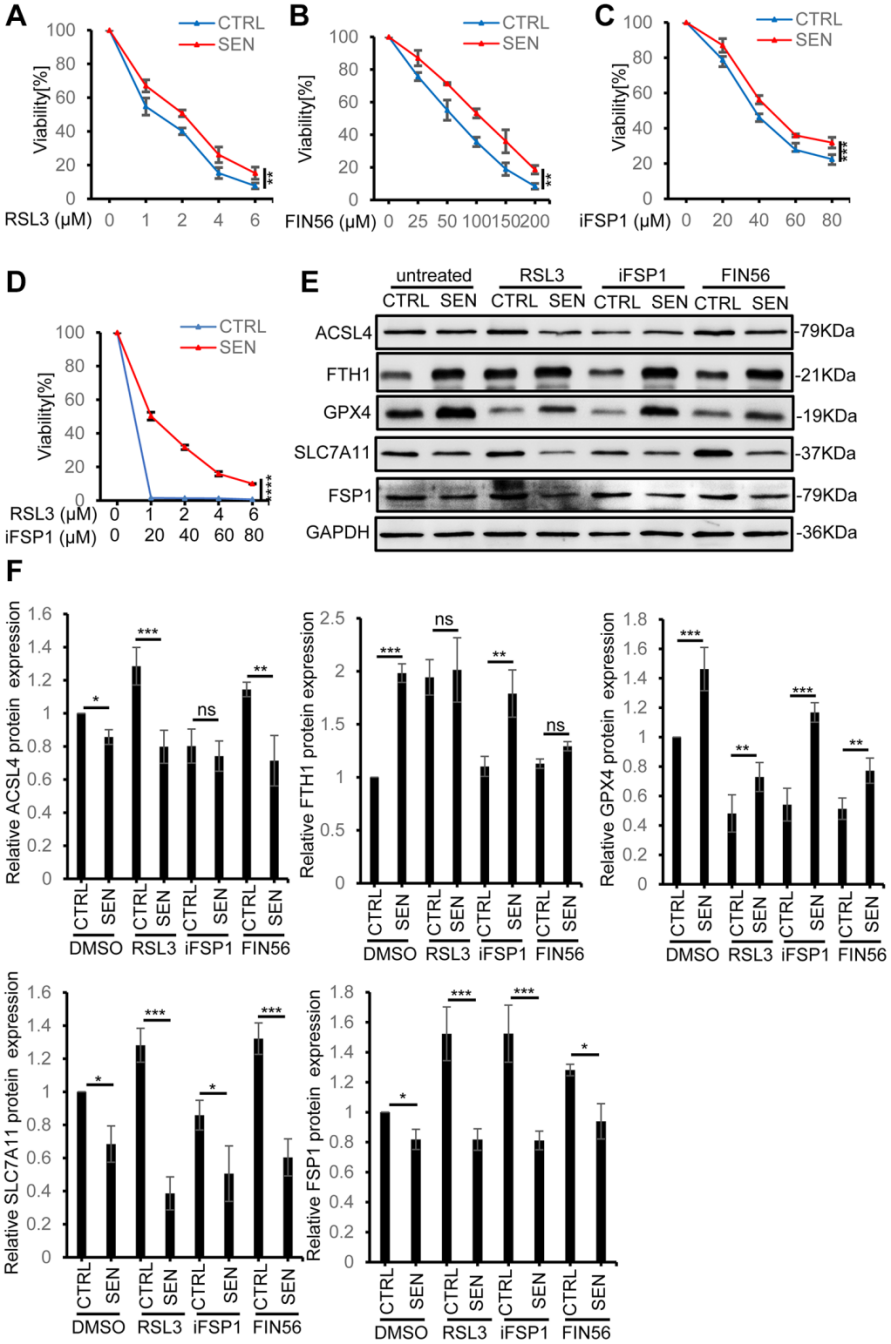
**Senescent HSkM cells are resistant to ferroptosis inducers.** (**A**–**C**) Senescent HSkM cells are more resistant to ferroptosis inducers RSL3, FSP1 and FIN56. The proliferating and senescent HSkM cells were treated with a series of concentrations of RSL3, FSP1 and FIN56 for 24 h, respectively. (**D**) Senescent cells are more resistant to the synergistical treatment of RSL3 and iFSP1. Cell viability was determined by CCK-8 assay. *P*-values were calculated by two-way ANOVA analysis, ^**^P < 0.01, ^***^P < 0.001. Data represented as mean ± SD. And three independent biological experiment repeats have been performed. (**E**, **F**) The expression of ferroptosis-related proteins in proliferating and senescent HSkM cells under RSL3, FIN56 and iFSP1 treatment. Immunoblot representative images (**E**) and quantification (**F**) of ACSL4, FTH1, GPX4, SLC7A11, FSP1 protein expression in normal and senescent HSkM cells after treatment with RSL3, iFSP1, FIN56, respectively. Each protein band intensity was measured and normalized to GAPDH and compared between proliferating and senescent cells. Data represented as mean ± SD. And three independent biological experiment repeats have been performed. *P*-values were calculated by one-way ANOVA analysis with post hoc Tukey, ^*^P < 0.05, ^**^P < 0.01, ^***^P < 0.001.

Considering the phenotype of high basal level of iron accumulation and lipid peroxidation, we supposed that senescent cells might stay at a pro-ferroptotic circumstance. Thus, we next tried to verify whether Dox-induced senescent HSkM cells are more susceptible to ferroptosis inducers. Given the slight increase of GPX4 expression in senescent cells, we firstly use a GPX4 inhibitor RSL3 to stimulate the ferroptosis in both normal and senescent HSkM cells. To our surprise, although senescent HSkM cells have high intrinsic intracellular iron accumulation, they are more resistant to RSL3 (Figure 3A). Subsequently, to validate this result, we used another GPX4 inhibitor FIN56 to treat normal and senescent cells. Senescent cells also exhibit more resistance to FIN56 (Figure 3B). Therefore, GPX4 should not be the key factor for senescent cells to resist endogenous ferroptotic stress. In addition, FSP1 functions as a GPX4-independent ferroptosis suppressor by catalyzing the regeneration of CoQ10 with NAD(P)H to reduce lipid peroxidation [29]. Here, we also found that senescent cells are resistant to the FSP1 inhibitor iFSP1, compared to proliferating cells (Figure 3C). To exclude the redundant effect of GPX4 and FSP1, we simultaneously treated senescent cells by RSL3 and iFSP1, and senescent HSkM cells still exhibit significant ferroptosis resistance as measured by CCK-8 (Figure 3D). Thus, we proposed that system Xc^−^-glutathione-GPX4 and FSP1-CoQ10-NADPH axis should not be the determining factors for senescent cells to resist basal ferroptotic stress.

To further analyze the causes for ferroptosis-resistance in senescent cells, we analyzed the key ferroptosis-related gene expression in normal and senescent HSkM cells after RSL3, FIN56 and iFSP1 treatment. As shown in Figure 3E, 3F, the individual ferroptosis inducer does not change the expression pattern of SLC7A11 and GPX4 in proliferating and senescent cells, and GPX4 expression increases and SLC7A11 decreases in senescent cells. FSP1 expression in senescent cells decreases upon different ferroptosis inducers compared to the proliferating cells. While FTH1, one subunit of ferritin, decreases under all the three drugs treatment, but the senescent cells still exhibit higher FTH1 expression compared to normal cells. On the contrary, ACSL4 increases in both normal and senescent HSkM cells with and without GPX4 inhibitors of RSL3 and FIN56 treatment, but did not show significant changes after iFSP1 treatment (Figure 3F).

Taken together, the high basal level of iron accumulation in senescent cells indeed activates the ferroptosis-related proteins in a different way with the ferroptosis inducers in proliferating cells, and also suggested that the ferritin-mediated ferritinophagy may play a key role in ferroptosis resistance in the senescent cells.

### Iron retardation in lysosome protects senescent HSkM cells from ferroptosis

**Figure 4:**
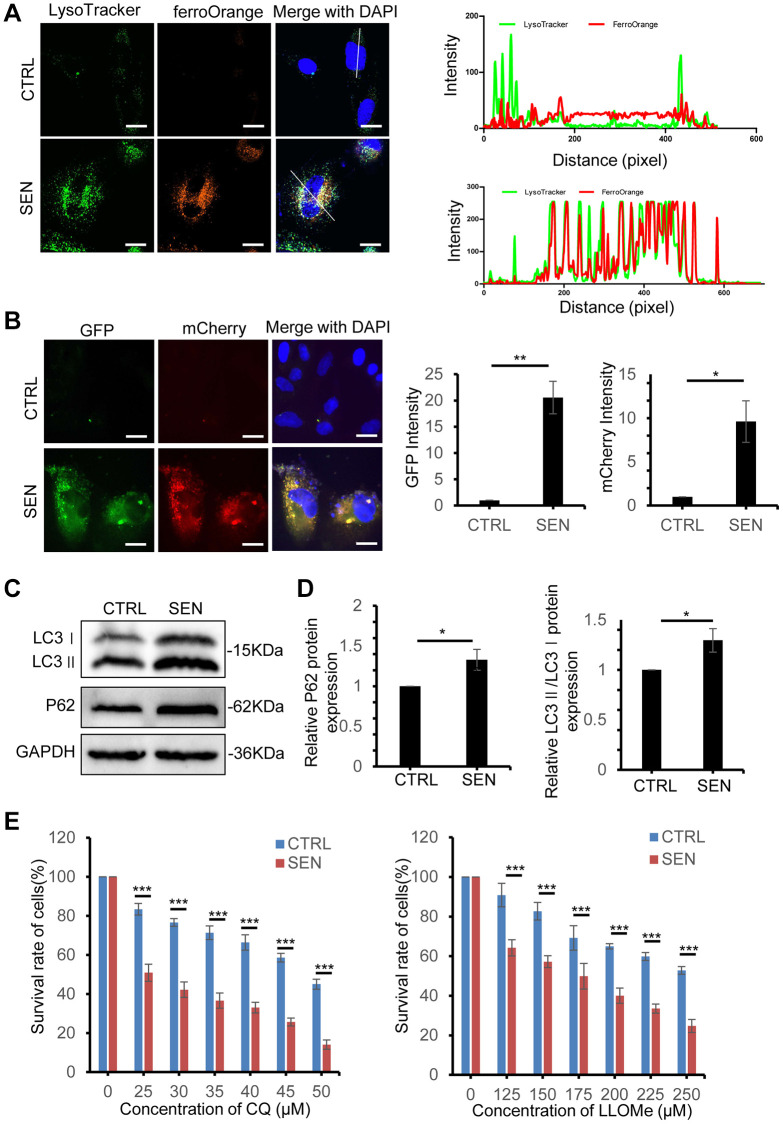
**Iron retention in lysosomes increases in senescent HSkM cells.** (**A**) Colocalization analysis of lysosome and Fe^2+^. Proliferating and senescent cells were co-stained with Lysosome dye (Lysotracker, in green) and Fe^2+^ probes (FerroOrange, in red), and observed by a confocal laser scanning microscopy. Representative live-cell fluorescence images were presented on the left panel, and intensity profile of lysosome and Fe^2+^ signals on the line across the cells were shown on the right panel. Scale bars, 20 μm. (**B**) Representative images of proliferating cells and senescent cells stably expressed the tandem reporter mCherry-GFP-LC3 to visualize the autophagic flux. Nuclei were stained with DAPI. Scale bars, 20 μm. (**C**) Immunoblot analysis of LC3 and p62 protein expression in normal and senescent HSkM cells. And three independent biological experiment repeats have been performed. (**D**) Quantification of the ratio of LC3-II: LC3-I and P62 protein expression. Each protein band intensity was measured and normalized to GAPDH and compared between proliferating and senescent cells. Data represented as mean ± SD. *P*-values were calculated by two-tailed unpaired student’s *t*-test, ^*^P < 0.05. (**E**) Dose-dependent toxicity assay of lysosomotropic agents chloroquine and LLOMe in proliferating and senescent HSkM cells. The proliferating and senescent cells were treated with a series of concentrations of CQ and LLOMe for 24 h, followed by the cell survival measurement by CCK-8 assay. Data represented as mean ± SD. And three independent biological experiment repeats have been performed. *P*-values were calculated by one-way ANOVA analysis with post hoc Tukey, ^***^P < 0.001.

Intracellular iron dramatically increases and forms spherical shape in senescent HSkM cells, meanwhile we also observed that both FTH1 and FTL are significantly increased (Figure 2A, 2D, 2E). Given that, we supposed that the overloaded iron in senescent cell might be enclosed and sequestered in senescent cell, which leads to the resistance to ferroptosis. To trace the loci of iron in cells, live-cell imaging of Lysotracker and FerroOrange probes co-stained cells manifested that most of the bright condensate FerroOrange foci are colocalized with lysosomes in senescent cells, but not in normal cells (Figure 4A). Thus, we postulated that the lysosome offered an enclosed space to trap iron and stop its release into cytoplasm in senescent cell, which would inhibit the ferroptosis induction and toxicity to other organelles, including mitochondria, endoplasmic reticulum, Golgi apparatus and others. Based on previous studies that the delivery of iron into lysosome relies on the ferritin-NCOA4 mediated autophagy process, we postulated the abnormal autophagy process during cell senescence [30]. To study the autophagic flux when cells come to senescence, we established HSkM cells with stably expressed mCherry-GFP-tagged LC3 and then induced cellular senescence, and both mCherry and GFP signals are very weak in normal cells, but senescent cells exhibit both strong GFP and mCherry signals (Figure 4B). In addition, both the expression of endogenous LC3 and P62 increase, and more LC3-I is converted into LC3-II in senescent cells (Figure 4C, 4D). The dysfunctional autophagic flux has been also observed in other stress-induced or replicative senescent cells [14].

To investigate the roles of lysosomes in senescent cells, we disrupted the functions of lysosomes by lysosomotropic agents chloroquine (CQ) and LLOMe (Leu-Leu-OMe) treatment. CQ treatment causes alkalinization of lysosome and inhibits autophagosome fusion with lysosomes [31]. And LLOMe is a well-characterized lysosomotropic agent to induce lysosomal rupture and leaky [32]. Fluorescent imaging of mCherry-GFP-tagged LC3 in EBSS, CQ and LLOMe treatment indicated that senescent HSkM cells show stronger mCherry and GFP punta signal to senescent cells. While cell survival assay indicates that the IC_50_ of CQ and LLOMe decrease by ~47% and 35% for senescence cell, respectively (Figure 4E and Supplementary Figure 3A). Next, we examined the loci of iron in senescent HSkM cells after CQ and LLOMe treatment, and results showed a significant decrease of aggregated iron foci in senescent cells (Supplementary Figure 3B). In addition, both CQ and LLOMe treatment increase the MDA production in senescent cells (Supplementary Figure 3C). We reduced the biogenesis of lysosome in both normal and senescent cells by knockdown of TFEB, a master gene for lysosomal biogenesis, and found that the survival of senescent cells is more dependent on the lysosome functions (Supplementary Figure 3E, 3F). Together, iron retention in lysosomes protect senescent cells from ferroptosis-induced cell death.

### Disruption of ferritinophagy process accelerates the death of senescent cells

**Figure 5:**
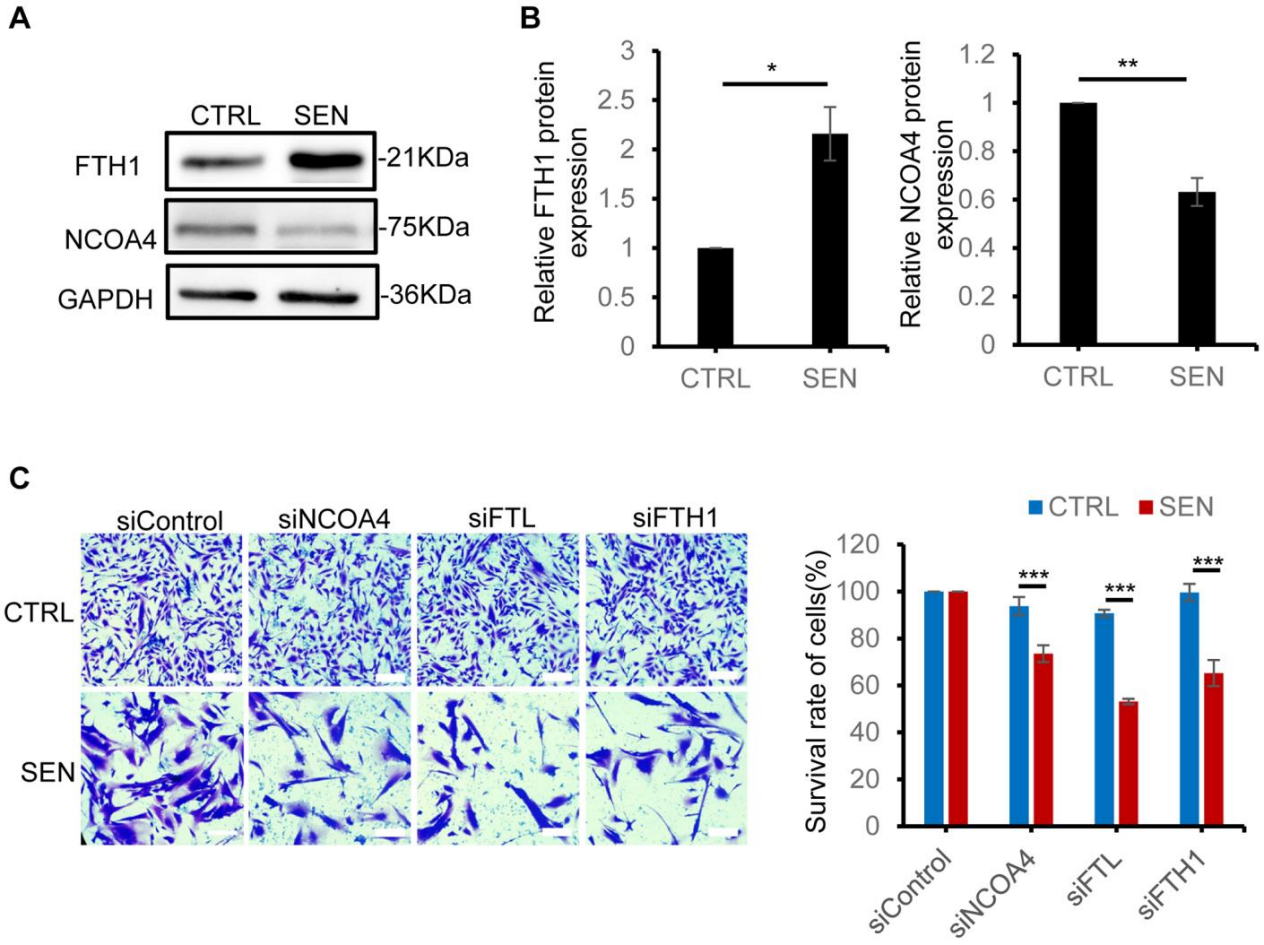
**Disruption of ferritinophagic process promotes senescent cell death.** (**A**) Immunoblot analysis of FTH1, NCOA4 protein expression in proliferation and senescent HSkM cells. (**B**) Quantification of the ratio of FTH1 and NCOA4 protein expression. Each protein band intensity was measured and normalized to GAPDH, compared between proliferating and senescent cells. Data were collected from three independent biological replicates, and represented as mean ± SD. *P*-values were calculated by two-tailed unpaired student’s *t*-test, ^*^P < 0.05. (**C**) FTH1, FTL and NCOA4 knockdown decreased the survival rate of senescent cells. The representative field images (left) and quantification of cell survival ratio (right). Cells were seeded on 24-well plate and the respective genes were knockdown by their specific siRNA for 48 h, followed by crystal violet staining. The cells were viewed by an inverted light microscope and counted the number of cells in different fields. The plate image is presented in Supplementary Figure 4E. Data were collected from three independent biological replicates, and represented as mean ± SD. *P*-values were calculated by one-way ANOVA analysis with post hoc Tukey, ^***^P < 0.001.

NCOA4-FTH1/FTL mediated ferritinophagy is a key process to transport cytosolic iron into lysosome by autophagy [30]. To investigate the role of ferritinophagy in senescent cells, we examined the ferritinophagy-associated protein expression in senescent HSkM cells. The increased amount of FTH1 in senescent cells enhanced the iron storage capacity (Figures 2D and 5A, 5B and Supplementary Figure 2). As for the selective ferritin cargo receptor NCOA4, its mRNA level does not change, but its protein level has shown a significant decrease by ~37% in senescent cells, which is probably caused by the enhanced lysosomal degradation of NCOA4 during cellular senescence (Figure 5A, 5B and Supplementary Figure 4A). Subsequently, to evaluate whether disrupting the process of ferritinophagy affects the survival of senescent cells, we knocked down FTH1, FTL and NCOA4 via siRNA in normal and senescent HSkM cells. The knockdown efficiency was tested by qPCR (Supplementary Figure 4B–4D). All the FTH1, FTL and NCOA4 knockdown comprised the survival of senescent cells, but not the normal cells (Figure 5C and Supplementary Figure 4E). To validate the Fe^2+^ transferring into lysosomes, we added extra iron into the medium to culture normal and senescent cells, and found only a very slightly increase of Fe^2+^ in the lysosomes of normal cells compared to senescent cells (Supplementary Figure 4F). Therefore, ferritinophagy-mediated iron transporting into lysosome is necessary to protect the senescent cell from excess iron accumulation.

### Lysosomal disruption inhibits the ferroptosis-resistance in senescent cells

**Figure 6:**
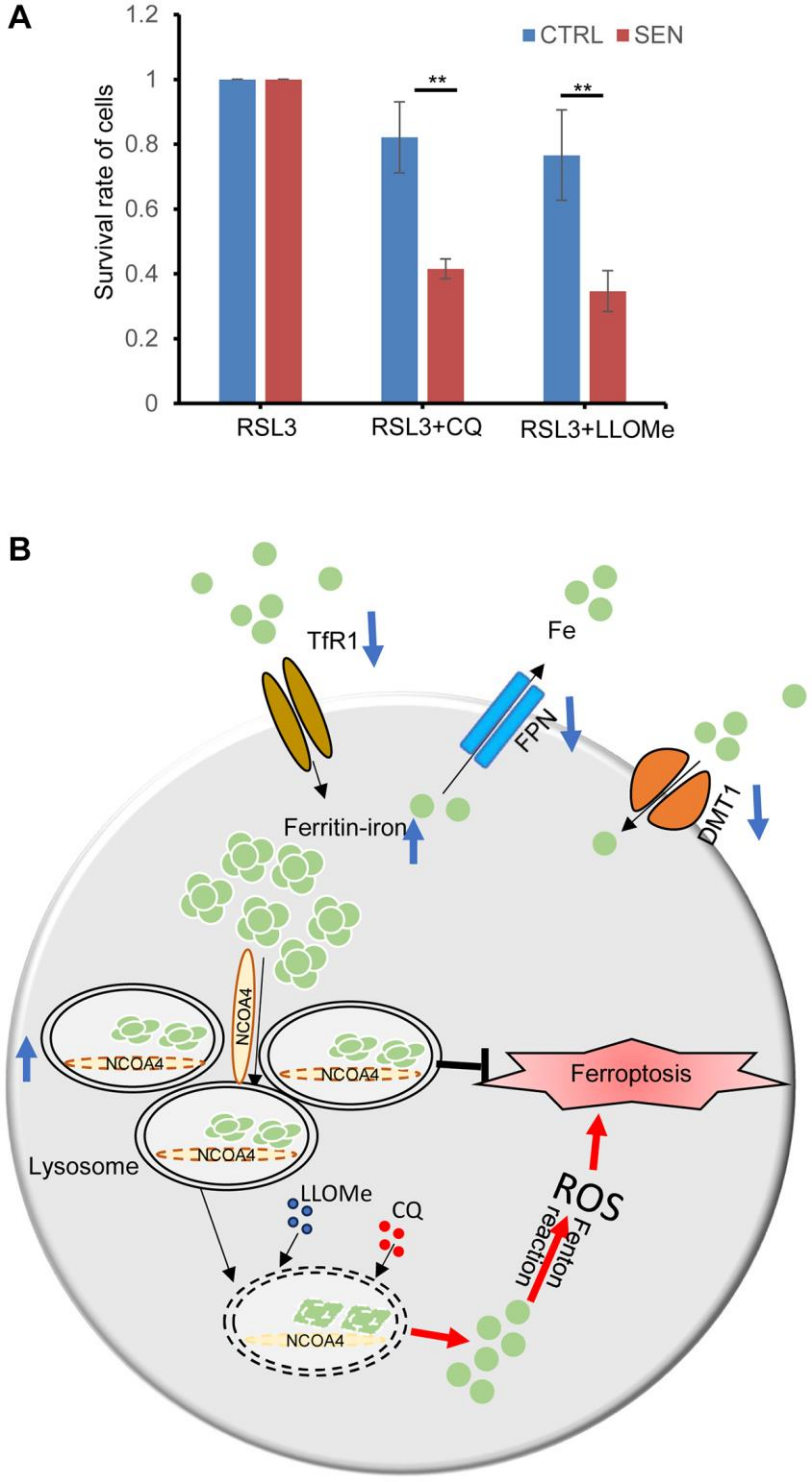
**Lysosome protects senescent cell from ferroptosis-induced cell death.** (**A**) CQ and LLOMe promote the senescent cell death under RSL3 treatment. Cells were treated with either combination of RSL3 and CQ or LLOMe for 12 h, and then cell viability was determined by CCK-8 assay. *P*-values were calculated by two-way ANOVA analysis, ^*^P < 0.05. Data represented as mean ± SD. And three independent biological experiment repeats have been performed. (**B**) A schematic model for cellular response to iron accumulation in senescent cells. In senescent cells, both the two-iron importer TfR1 and DMT1 expression decreases, probably due to feedback repression of iron accumulation. And iron exporter FPN1 also decreases to reduce the iron efflux from cells. Subsequently the accumulated intracellular Fe^2+^ is bound by the ferritin protein nanocages, and transferred into lysosomes via NCOA4-mediated ferritinophagy. Besides, the number of lysosomes increases, and their size are enlarged in senescent cells, which provides enough space to store the excessive iron. Once the structures and/or functions of lysosome of senescent cells are broken by CQ or LLOMe, the toxic contents of lysosomes would be released, including the labile Fe^2+^ and others, which leads to ferroptosis-induced cell death.

Senescent HSkM cells are resistant to the ferroptosis inducers, and the endogenous excessive iron is sequestered in the lysosomes. Thus, we simultaneously treated cells with CQ and LLOMe to inhibit the functions of lysosome in senescent cells, and found that senescent cells become more sensitive to ferroptosis inducer RSL3 (Figure 6A). Thus, the lysosome is a promising senolytic target to overcome ferroptotic resistance and cure age-related diseases.

## DISCUSSION

Cellular senescence is a key cause of tissue aging and also involves in the progression of organismal aging and the development of age-related diseases [33]. Discerning and selectively cleaning of senescent cells in tissues have been reported as an effective therapeutic strategy to improve the healthy life span [34]. Here, we used the Dox-induced senescent HSkM cell model, and confirmed that iron dramatically increased and lipid peroxidation also elevated in the induced senescent cells (Figure 2A, 2B). We postulated that the iron increase might mainly result from the decreased expression of iron exporter FPN. While iron accumulation and lipid peroxidation have been well recognized as the two major biochemical events during ferroptosis. Surprisingly, Dox induced senescent HSkM cells are more resistant to various kinds of ferroptosis inducers compared to the proliferating cells (Figure 3A–3D). Such ferroptosis resistance also occurs in other stimuli induced senescent cells [14]. We further proved that ferroptosis resistance in senescent cells is independent of the GPX4- or FSP1-mediated cellular protection pathway (Figure 3D). Iron increase in senescent cells is accompanied by the robustly elevated expression of ferritin, which can be transported into the lysosomes via NCOA4-mediated ferritinophagic process (Figures 4A and 5A–5C). We traced the iron location via FerroOrange probe staining and demonstrated that the excess iron in senescent cells has been trapped into the lysosomes (Figure 4A). The two lysosomotropic agents, CQ and LLOMe, target lysosomes and release the iron and other toxicities to cytoplasm, leading to cell death (Figure 6B). Thus, the lysosomal targeting strategy confers valuable choice for the development of great potent drugs to overcome the ferroptosis resistance and promote the removal of the senescent cells.

In particular, plenty of evidence indicates that iron is an essential trace element almost in all living organisms to facilitate various cellular biological processes. The major role of iron is oxygen transport, and erythrocyte hemoglobin contains about 80% of total body iron. Thus, iron deficiency is the main cause of anemia world widely [35]. Nonheme iron deposition in body exists in multiple forms, such as labile iron, lipofuscin and ferritin. And macrophages and hepatocytes also store a lot of iron and regulate the iron homeostasis of body iron [36]. Besides, iron could cross the blood-brain barrier, and the accumulation and distribution changes of iron in brain are closely associated with various neurodegenerative diseases, including Alzheimer’s and Parkinson’s diseases [37]. Emerging evidence indicates that dysregulation of iron deposition-associated proteins during aging leads to the abnormal cellular processes during cell senescence. And excessive nonheme iron has been reported to lead to lifespan hazard in mammals, drosophila and nematode during aging [38, 39], and therefore the detailed relationship between the iron accumulation and aging has gained attention in the field of gerontological research in recent decades [40]. As reported, dysregulation of cellular iron concentration is susceptible to multiple age-related diseases such as cardiovascular disease, cancers, and neurodegenerative diseases [41–43]. Thus, iron accumulation is a hallmark of aging and cellular senescence, which can be addressed as physiological requirement for iron such as iron-dependent protein activities, oxygen transfer, or pathological incompetence of iron regulatory mechanisms of the dysfunctional flux of iron import, storage and efflux [14, 44]. The cellular toxicity of excessive iron is due to the iron-dependent ROS production via Fenton reaction, mediating cell death from ferroptosis [45]. Free radical theory is supposed as the basic mechanism of aging by Harman in 1956 [46]. Consistent with our results, senescent cells exhibit dramatic accumulation of oxidative lipid and MDA production (Figure 2B, 2C). Oxidative stress is a principal factor inducing ferroptosis by iron dependent lipid peroxidation. Given that, we speculated that the sources of ROS at least partially come from iron-mediated ferroptosis pathway, also come from other biological pathways, such as dysfunctional mitochondria and others [47, 48].

Based on our results, we proposed that excessive iron is sequestered in lysosome to inhibit ferroptosis, which protecting senescent cells from ferroptosis-mediated cell death (Figure 4). Hence, we revisited lysosomal changes of senescent cells. The characteristics of lysosomes in senescent cells have been documented as remarkably increased lysosomal amounts, higher pH, damaged membrane and decreased proteolytic capacity etc., [49]. We also observed an increase of both the endogenous LC3 and P62 expression, and robust elevated GFP-mCherry-LC3 puncta in senescent cell (Figure 4C). Thus, the autophagy-lysosome pathway in senescent cells is apparently activated. Given that both the GFP and mCherry signal of GFP-mCherry-LC3 puncta increase under both fed, starve (EBSS treatment), CQ and LLOMe treatment in senescent cell, we speculated the increase of total autophagic vacuoles, including the autophagosomes and lysosomes (Supplementary Figure 3D). Although the gross of lysosomes has greatly increased, its capability of proteolysis shows less active. The proteomic analysis indicates that the lysosomes amass much more non-lysosomal substrates in senescent cells, which might be caused from the accumulation of undegraded cargoes due to the decline of proteolytic activity [49, 50]. In this respect, the enlarged lysosome in senescent cells can be considered as a storeroom to trap and store the extra iron and its associated proteins in senescent cells.

Both the CQ and LLOMe, two lysosome-damaging agents, can interfere the structure and functions of lysosomes by different mechanisms, namely deacidifying the lysosomal lumen and permeabilizing lysosome membrane [31, 32]. Our results indicates that senescent HSkM cells are more sensitive to these two drugs (Figure 4E and Supplementary Figure 3A). It is likely that the damaged lysosomal membrane makes the lysosome more sensitive to lysosomotropic agents in senescent cells. Subsequently, lysosomal cargoes would flow into cytoplasm, resulting in cell death. In our studies, the lysosomotropic agents increase the iron efflux from lysosomes and overcome the ferroptosis resistance (Figure 6A).

In conclusion, we found that the ferroptosis-resistance of senescent cells is due to the autophagy-mediated iron storage in lysosome. And lysosome-targeting agents are potent candidates for senolytic drugs. Iron accumulation-mediated ferroptosis contributes to ROS increase when lysosome dysfunction occurs in senescent cells, but the following cellular processes that influence other organelles, including the mitochondria and lipid droplets and finally lead to senescent cell death, need to be further investigated.

## Supplementary Materials

Supplementary Figures
