# Correction for: Iron retardation in lysosomes protects senescent cells from ferroptosis

**DOI:** 10.18632/aging.206052

**Published:** 2024-07-31

**Authors:** Yujing Feng, Huaiqing Wei, Meng Lyu, Zhiyuan Yu, Jia Chen, Xinxing Lyu, Fengfeng Zhuang

**Affiliations:** 1 School of Laboratory Animal and Shandong Laboratory Animal Center, Shandong First Medical University and Shandong Academy of Medical Sciences, Jinan, Shandong, China; 2 School of Clinical and Basic Medical Sciences, Shandong First Medical University and Shandong Academy of Medical Sciences, Jinan, Shandong, China; 3 Biomedical Research College and Shandong Medicinal Biotechnology Centre, Shandong First Medical University and Shandong Academy of Medical Sciences, Jinan, Shandong, China; 4 School of Radiology, Shandong First Medical University and Shandong Academy of Medical Sciences, Taian, Shandong, China

**Keywords:** iron accumulation, senescent cells, lysosome, ferroptosis, ferritinophagy

**This article has been corrected:**The corresponding author Fengfeng Zhuang is now affiliated with the University of Sanya and asked that his current address be updated. In addition, the authors forgot to mention an additional source of funding, the Major Basic Research Project of the Shandong Natural Science Foundation (ZR2020ZD12), and would like to correct this mistake.

The updated list of the affiliations and Funding are provided below.

Yujing Feng^1,*^, Huaiqing Wei^3,*^, Meng Lyu^2^, Zhiyuan Yu^2^, Jia Chen^4^, Xinxing Lyu^2^, Fengfeng Zhuang^1,5^

^1^School of Laboratory Animal and Shandong Laboratory Animal Center, Shandong First Medical University and Shandong Academy of Medical Sciences, Jinan, Shandong, China

^2^School of Clinical and Basic Medical Sciences, Shandong First Medical University and Shandong Academy of Medical Sciences, Jinan, Shandong, China

^3^Biomedical Research College and Shandong Medicinal Biotechnology Centre, Shandong First Medical University and Shandong Academy of Medical Sciences, Jinan, Shandong, China

^4^School of Radiology, Shandong First Medical University and Shandong Academy of Medical Sciences, Taian, Shandong, China

^5^Current address: Health Medical School, University of Sanya, Sanya, Hainan, China

^*^Equal contribution

