# Supplementary material for: Iron retardation in lysosomes protects senescent cells from ferroptosis
Source: Aging (Albany NY). 2024 Apr 26;16(9):7683–703. doi: 10.18632/aging.205777 (PMC11131988; doi:10.18632/aging.205777)
Supplement: Supplementary Figures [file aging-16-205777-s001.pdf]

SUPPLEMENTARY FIGURES

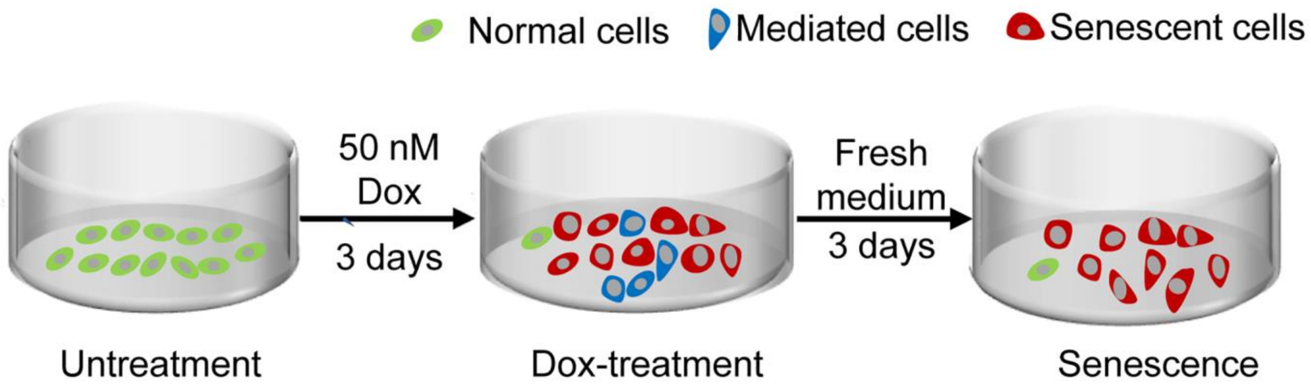

Supplementary Figure 1. Scheme of the buildup of Dox-induced senescent HSkM cell model. The proliferating HSkM cells were treated with 50 nM Dox for 3 days, then the drug was removed and fresh DMEM was used to continue to culture the cells for another 3 days to exclude the direct toxicity of Dox for cells. The characteristics of senescent HSkM cells have been described as shown in Figure 1A–1F.

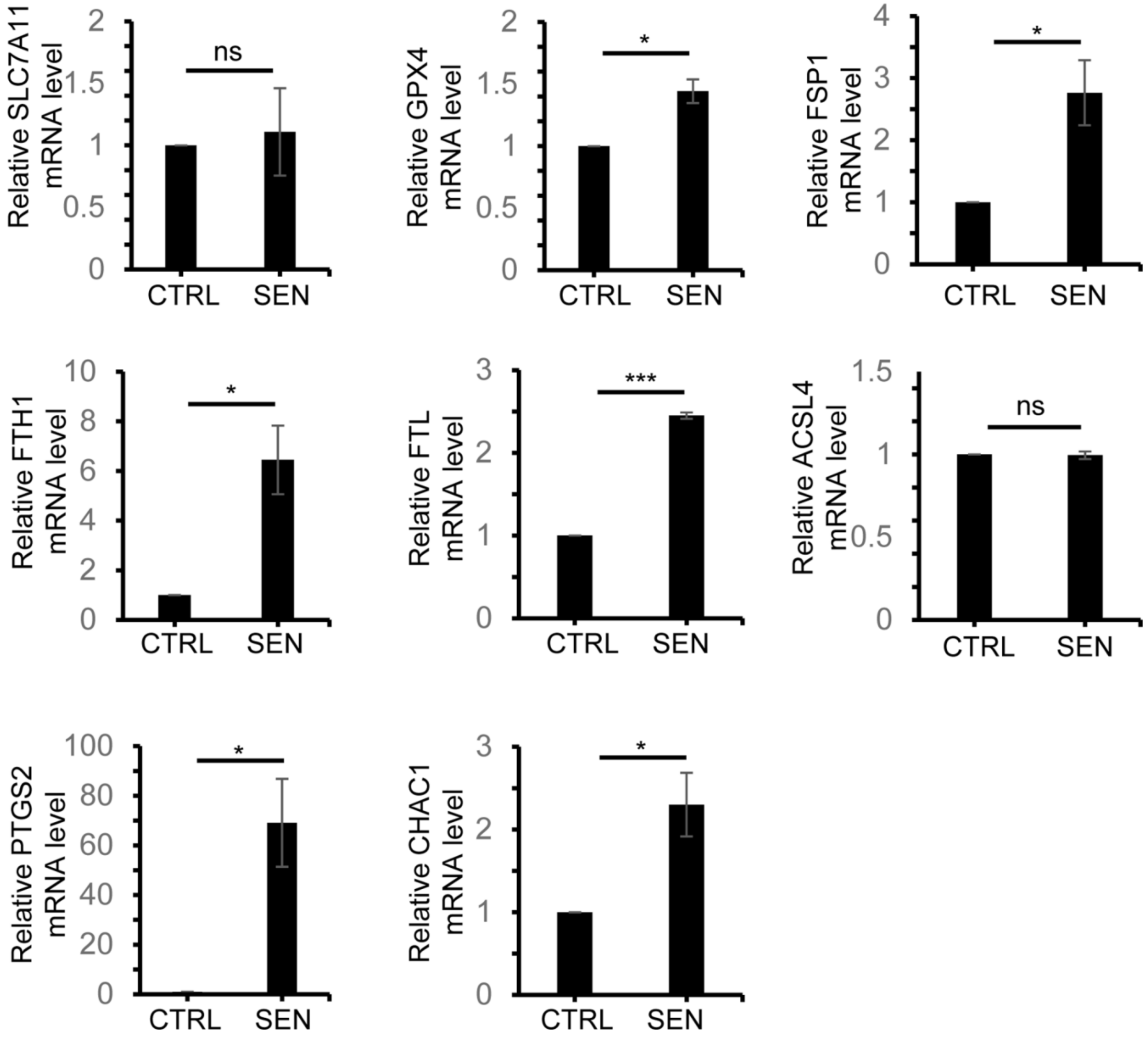

Supplementary Figure 2. Transcriptional analysis of the ferroptosis-related gene expression in senescent HSkM cells. mRNA expression of SLC7A11, GPX4, FSP1, FTH1, FTL, ACSL4, PTGS2 and CHAC1 in proliferating and senescent HSkM cells are included. Three biological experiment repeats have been performed. *P*-values were calculated by two-tailed unpaired student's *t*-test, $^{*}P < 0.05$, $^{***}p < 0.001$. Data represented as mean ± SD.

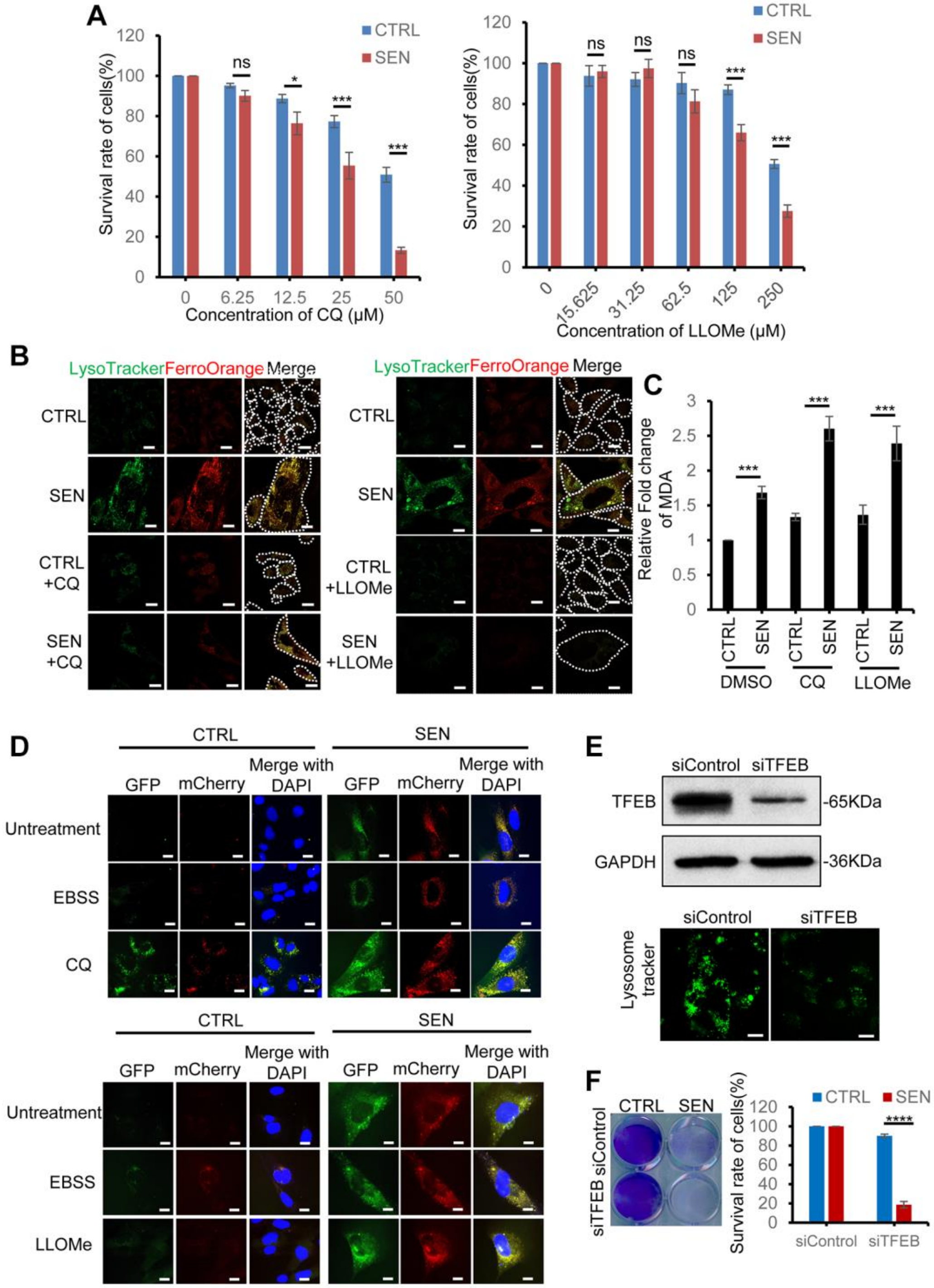

Supplementary Figure 3. CQ and LLOMe inhibit the accumulation of iron in the lysosome of senescent HSkM cells. (**A**) Cell survival analysis of proliferating and senescent HSkM cells with a variety of concentration of CQ (0-50 μM) (Left) and LLOMe (0-250 μM) (right) treatment. The proliferating and senescent cells were treated with a series of concentrations of CQ and LLOMe for 24 h, followed by cell survival measurement by CCK-8 assay. Data represented as mean ± SD. And three independent biological repeats have been performed. *P*-values were calculated by one-way ANOVA analysis with post hoc Tukey, $^{*}P < 0.05$, $^{***}P < 0.001$. (**B**) CQ and LLOMe treatment decrease the Fe^{2+} accumulation in lysosomes of senescent cells. Both the proliferating and senescent cells were treated with CQ and LLOMe for 12 h, followed by the fluorescence probes of FerroOrange and lysotracker incubation for 30 min. The outline of cells was marked with white dash lines. Scale bar: 20 μm. (**C**) CQ and LLOMe treatment increases the MDA production. Both the proliferating and senescent cells were treated with CQ and LLOMe for 24 h, followed by MDA measurement with kit. Three independent biological repeats have been performed. *P*-values were calculated by one-way ANOVA analysis with post hoc Tukey, $^{***}P < 0.001$. (**D**) CQ and LLOMe treatment does not alter the enhanced Cherry-GFP-LC3 aggregation. Stably expressed tandem reporter mCherry-GFP-LC3 in proliferating and senescent HSkM cells were treated with EBSS, CQ or LLOMe for 6 h. Nuclei were stained with DAPI. Scale bars, 20 μm. (**E**) Knockdown of TFEB

decreases the biogenesis of lysosomes. The knockdown efficiency of TFEB is tested by Western blot (upper panel) and the lysosomes are stained by lysotracker (lower panel). Scale bars, 10 μm. (**F**) Reduction of lysosome biogenesis decreases the survival of senescent cells. Crystal violet staining analysis of overall scanned plate for cell survival in normal and senescent HSkM cells with TFEB knockdown (left panel) and the quantification (right panel).

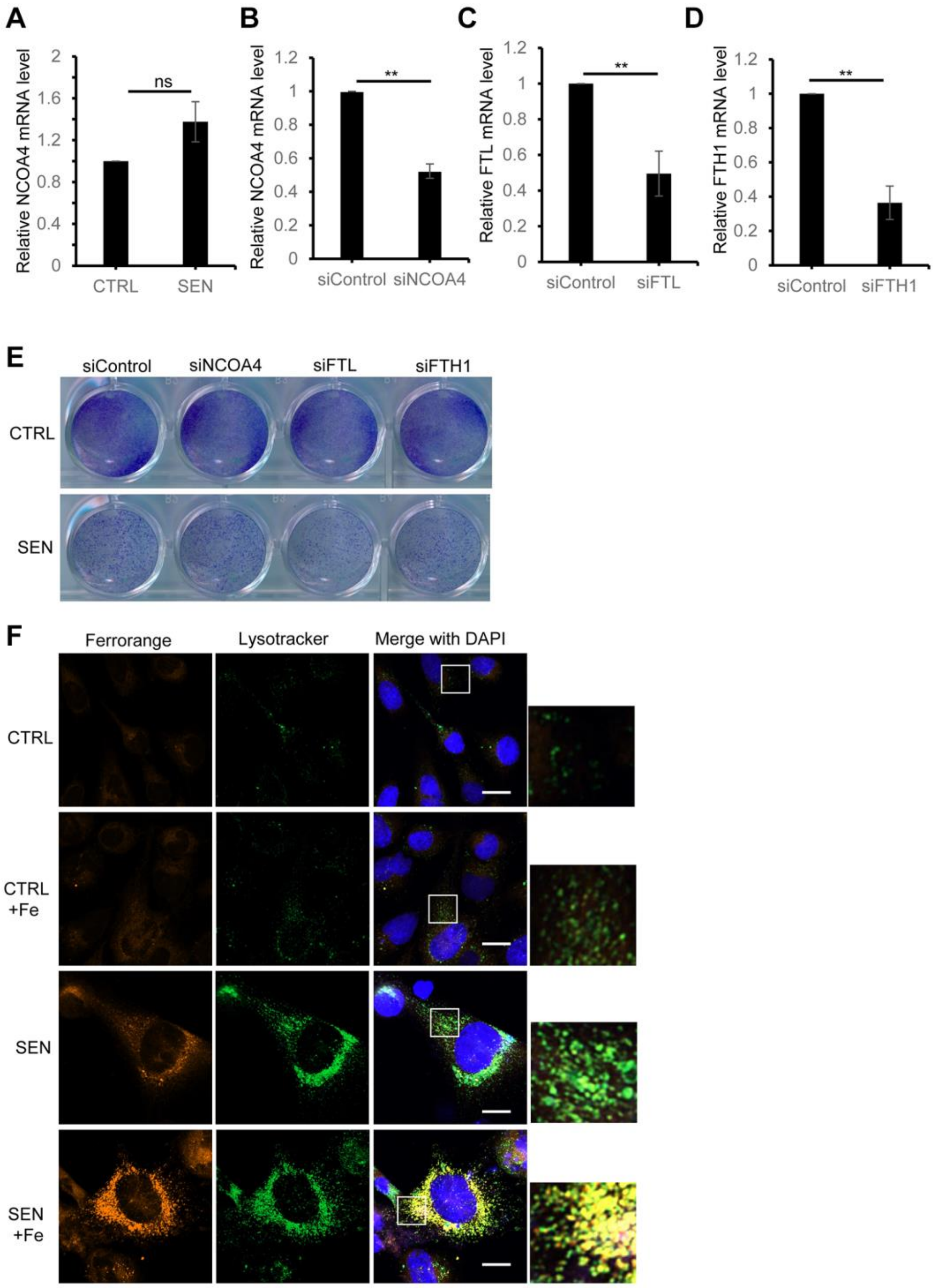

Supplementary Figure 4. Ferritinophagy contributes to the survival of senescent HSkM cells. (**A**) Transcriptional analysis of NCOA4 in senescent cells by qPCR. (**B**–**D**) The knockdown efficiency of NCOA4, FTL and FTH1 was determined by qPCR. (**E**) Crystal violet staining analysis of overall scanned plate for cell survival in normal and senescent HSkM cells with NCOA4, FTL and FTH1 knockdown by their specific siRNA. (**F**) The extra iron addition slightly increases the Fe^{2+} concentration in normal cells compared to senescent cells. Nuclei were stained with DAPI. Boxed areas are amplified in inserts to indicate Fe^{2+} and lysosome colocalization. Scale bars, 20 μm. All the experiments have been performed for at least three biological repeats. *P*-values analysis for qPCR were calculated by two-tailed unpaired student's *t*-test, $^{**}P < 0.01$. Data represented as mean ± SD.
